# Assessment of Superparamagnetic Iron Oxide Nanoparticle Poly(Ethylene Glycol) Coatings on Magnetic Resonance Relaxation for Early Disease Detection

**DOI:** 10.1109/OJEMB.2020.2989468

**Published:** 2020-05-04

**Authors:** Cari L. Meisel, Polly Bainbridge, Robert V. Mulkern, Dimitrios Mitsouras, Joyce Y. Wong

**Affiliations:** ^1^ Department of Biomedical EngineeringBoston University 1846 Boston MA 02215 USA; ^2^ Department of RadiologyHarvard Medical School 1811 Boston MA 02115 USA; ^3^ Department of RadiologyBoston Children's Hospital 1862 Boston MA 02115 USA; ^4^ Brigham and Women's Hospital 1861 Boston MA 02115 USA; ^5^ Department of Radiology and Biomedical ImagingUniversity of California 8785 San Francisco CA 94143 USA; ^6^ Department of Radiology ServiceSan Francisco Veterans Affairs Medical Center 19980 San Francisco CA 94121 USA; ^7^ Department of Biomedical Engineering and Division of Materials Science and EngineeringBoston University 1846 Boston MA 02215 USA

**Keywords:** Magnetic resonance imaging (MRI), magnetic relaxation, poly(ethylene glycol), SPIONs

## Abstract

*Objective*: Superparamagnetic Iron Oxide Nanoparticles (SPIONs) are widely researched as contrast agents in clinical magnetic resonance imaging (MRI). SPIONs are frequently coated with anti-biofouling substances such as poly(ethylene glycol) (PEG) to prevent protein deposition and improve circulation time *in vivo*. The aim of this study is to optimize SPION MR properties with respect to physicochemical properties of the core SPION and the polymeric coating to better understand the interaction of these parameters and the efficacy of the designed agent. *Methods*: We used different methods of chemical attachment of a polymer, polymer chain length, and polymer coating density and examined their effects on the MR relaxivities of SPIONs. *Results*: These studies indicate that the chemical composition and, in particular, the hydrophobicity/hydrophilicity of the chemical group linking PEG chains to a SPION core may play a larger role in the resulting MR relaxivities than other variable properties such as SPION core size and PEG chain length. *Conclusions*: The method of SPION fabrication and chemical composition of the coating play a significant role in the MR relaxivities of the resulting particles. These results should be considered in the fabrication of particles for clinical purposes, particularly when optimization of the MR relaxivities is desired.

## I. Introduction

Superparamagnetic iron oxide nanoparticles (SPIONs) are used as contrast agents for clinical magnetic resonance imaging (MRI) [1]–[3]. SPION variability in terms of iron core size, surface functionalization, and targeting moieties makes them potentially well-suited for detection of a wide array of diseases [4]–[9]. SPIONs hold specific advantages over widely-used gadolinium-based MR contrast agents: each nanoparticle contains thousands of iron atoms and approaches saturation magnetization in magnetic fields typically used for MR imaging, i.e., each nanocrystal can generate signal contrast several orders of magnitude higher than gadolinium chelates [10]. Additionally, SPIONs have demonstrably lower toxicity *in vivo* than gadolinium-based contrast agents [11]–[15]. These two features make SPIONs particularly well-suited for early detection or diagnosis of diseases ranging from cancer to atherosclerosis.

Upon injection into the bloodstream, SPIONs immediately encounter a high-protein environment where adsorption of various proteins onto the SPION surface can ultimately lead to rapid clearance, reducing their effectiveness as a contrast agent [16]–[18]. A frequently used strategy to prevent protein deposition is to coat SPIONs with an antibiofouling layer such as poly(ethylene glycol) (PEG) [14], [19]. PEG is thought to display good resistance against nonspecific protein adsorption in large part due to its extensive hydrogen bonding with water [20]. SPIONs are often coated with PEG in addition to targeting moieties such as antibodies, peptides, or aptamers, [7], [21] and have been thoroughly reported in the literature for *in vivo* imaging of early disease markers.

One challenge of using molecular-targeted contrast agents at early disease stages is that molecular markers are usually present in small quantities [22], [23]. Detecting small quantities of these markers is challenging in MRI even with the benefit of SPION-enhanced contrast; thus maximal MR signal is needed to improve disease detection sensitivity. Clinically, increasing MR relaxivities can yield several benefits, including a greater signal-to-noise ratio and therefore increased resolving power that may be particularly beneficial in applications such as early disease detection. Moreover, increased MR relaxivities may allow lower administered doses to yield equivalent signal-to-noise ratios, meaning that agents with increased MR relaxivities may carry a cost and/or safety benefit to patients.

 Few previous studies have comprehensively examined optimization of SPION MR properties with respect to physicochemical properties.

SPIONs create contrast in MR imaging by affecting two properties of nearby water protons: [24] T_1_ (spin-lattice relaxation time) and T_2_ (spin-spin relaxation time). The corresponding relaxivities r_1_ and r_2_ are in part determined by translational diffusion of water molecules in the inhomogeneous magnetic field caused by the SPIONs [10]. Although SPIONs impact r_1_ and r_2_, they are primarily used as T_2_ contrast agents because of their larger r_2_ relaxivities [25], [26]. Two design parameters of SPIONs and their chemical coatings known to impact relaxivities are (1) iron oxide core (size and material), and (2) polymer coating surrounding the SPION core, which includes coating density (i.e., polymer chains per nm^2^), thickness (i.e., polymer chain length), and chemical composition.

Some prior work has explored the first aspect of SPION contrast agents. It is known that for a fixed total amount of iron in the core, r_2_ relaxivity of SPIONs increases with magnetization and total core size (i.e., iron oxide plus other elements that may be added to the core) [4], [27]. Altering SPION magnetization by doping iron oxide with other magnetic elements (e.g., nickel, cobalt, and manganese) leads to enhancement of measured relaxivities [5], [27], [28].

However, effects of changes in polymer coating in terms of density, thickness, and chemical composition on relaxivities of the resulting SPION agents has not been well-characterized to date. Of the limited prior studies, none have controlled for or measured coating density, which is thought to have a direct effect on the resultant relaxivities [15], [p. 2], [29]. The importance of understanding how altering the SPION core coating may impact MR signal is in part due to the fact that the strength of the magnetic field surrounding the SPION core diminishes rapidly with distance; thus, the strongest part of the magnetic field surrounding the SPION often falls within the polymer coating [29], [30]. Moreover, SPION functionalization using different coating schemes, particularly in terms of method of chemical attachment of the coating to the core and polymer chain length, may affect their MR properties even if the coating density remains constant. The aim of the present study is to determine effects of different methods of chemical attachment of a polymer, polymer chain length, and polymer coating density on SPION MR relaxivities, thereby contributing to a better understanding of the interaction of these parameters and the efficacy of the designed agent. These results will aid in optimization of SPION MR properties with the aim of improving SPION contrast agents intended for clinical imaging, especially in early disease detection.

## II. Results

**Figure 1.**
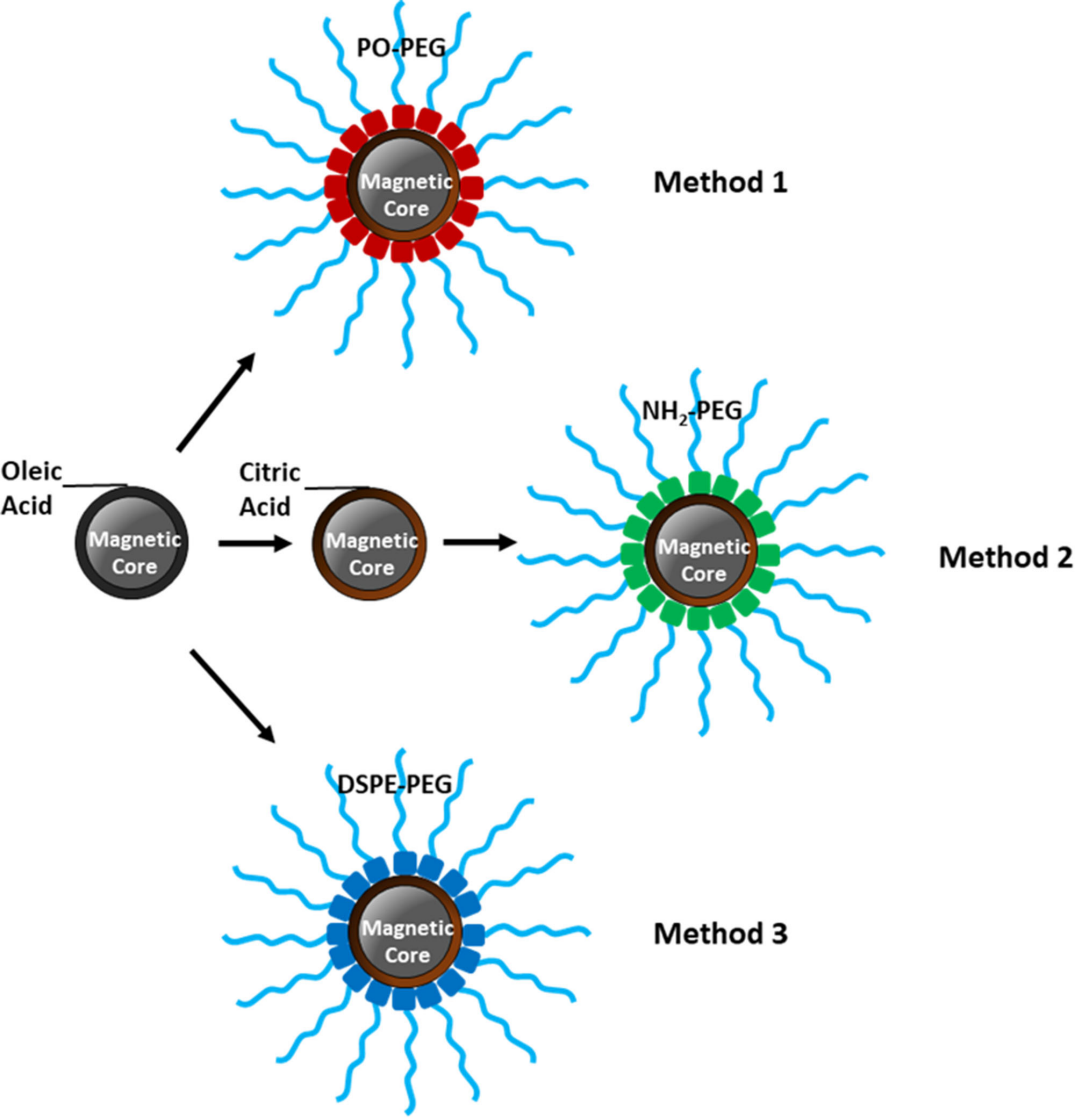
Schematic of particle fabrication. Three methods were used: (1) direct PEGylation via ligand exchange with phosphine oxide (PO)-PEG, (2) ligand exchange to coat particles with citric acid, followed by covalent addition of NH_2_-PEG via EDC/NHS chemistry, and (3) PEGylation via hydrophobic interactions between oleic acid on the SPION surface and 1,2-Distearoyl-sn-glycero-3-phosphoethanolamine (DSPE) residues on DSPE-PEG. For each method of fabrication, at least 3 different PEG chain lengths were added to different particle samples to observe effects of polymer chain length on resulting MR relaxivities. Tested PEG chain lengths were: (1) for PO-PEG SPIONs – 1000, 2000, and 5000 Da, (2) for NH2-PEG SPIONs – 1000, 2000, 3400, and 5000 Da, and (3) for DSPE-PEG SPIONs – 1000, 2000, 5000 Da. In total, 20 unique SPIONs were fabricated, characterized, and tested.

Both 4nm and 10nm SPION cores were successfully functionalized using 3 different fabrication schemes (Fig. 1).

Dynamic light scattering (DLS), zeta potential measurements, and thermogravimetric analysis (TGA) were used to characterize and confirm success of SPION fabrication for each type of SPION produced (Table 1).

**TABLE 1.** Summary of Characterization Results for all SPION Variants Produced. Results are Represented as Mean ± Standard Deviation, n = 3. Effective Diameter was Measured Using DLS, Zeta Potential Was Measured on the Same Machine, and Surface Density was Calculated From TGA Results. All PEG Chains Were Linear in Conformation

**Diameter (nm)**	**PEGylation Method**	**PEG Size (kDa)**	**Effective Diameter (nm)**	**Zeta Potential (ζ) (mV)**	**Surface Density (PEG chains/nm^2^)**
4	PO-PEG (Method 1)	1000	18.66 ± 4.14	-14.75 ± 1.58	0.56 ± 0.06
		2000	22.67 ± 3.21	-5.63 ± 6.13	0.82 ± 0.09
		5000	22.86 ± 2.54	-1.62 ± 4.21	0.93 ± 0.23
	NH_2_-PEG (Method 2)	1000	14.79 ± 3.74	-19.46 ± 1.71	0.68 ± 0.13
		2000	18.04 ± 4.24	-15.89 ± 1.11	0.93 ± 0.04
		3400	38.46 ± 6.16	-10.85 ± 1.29	0.87 ± 0.06
		5000	20.03 ± 4.42	-8.93 ± 1.21	0.86 ± 0.15
	DSPE-PEG (Method 3)	1000	64.24 ± 16.19	-23.55 ± 13.85	0.70 ± 0.05
		2000	82.40 ± 26.49	-18.36 ± 23.57	0.82 ± 0.05
		5000	52.98 ± 11.54	22.17 ± 19.41	1.13 ± 0.11
10	PO-PEG (Method 1)	1000	40.69 ± 5.42	-6.30 ± 1.29	0.41 ± 0.10
		2000	42.71 ± 7.45	-13.35 ± 3.16	0.67 ± 0.13
		5000	45.93 ± 5.47	-8.94 ± 0.89	0.66 ± 0.18
	NH_2_-PEG (Method 2)	1000	30.22 ± 5.46	-22.79 ± 2.75	0.35 ± 0.31
		2000	27.33 ± 5.17	-13.79 ± 1.84	0.62 ± 0.08
		3400	31.12 ± 5.57	-20.67 ± 1.39	0.67 ± 0.03
		5000	41.21 ± 6.35	-13.54 ± 1.65	0.60 ± 0.14
	DSPE-PEG (Method 3)	1000	58.07 ± 15.21	-31.14 ± 31.40	0.69 ± 0.00
		2000	75.45 ± 28.67	-16.10 ± 33.98	0.69 ± 0.01
		5000	55.49 ± 11.70	-3.61 ± 16.48	0.77 ± 0.27

In phosphine oxide (PO)-PEG SPIONs, nanoparticle effective diameter appears to increase as PEG chain length increases, yet these differences were not statistically significant across multiple batches (p > 0.05). For 1,2-Distearoyl-sn-glycero-3-phosphoethanolamine (DSPE)-PEG SPIONs, the 5k PEG samples had a smaller effective diameter than either the 1k or the 2k PEG, but these differences were not significant (p > 0.05). This trend was also observed for 4nm NH_2_-PEG SPIONs, with 3.4k NH_2_-PEG SPIONs being significantly larger (p < 0.05) than 1k, 2k, and 5k NH_2_-PEG SPIONs. For 10nm NH_2_-PEG SPIONs, this did not appear to be the case: 5k NH_2_-PEG SPIONs were significantly larger than 1k, 2k, and 3.4k NH_2_-PEG SPIONs. For both PO-PEG and NH_2_-PEG SPIONs, the effective diameters of the 4nm samples were significantly smaller (p < 0.05) than the equivalent 10nm samples. For DSPE-PEG SPIONs, this was not the case. Finally, it can be observed for all samples that for a given core size and PEG chain length, the effective diameter observed between different PEGylation methods was such that NH_2_-PEG SPIONs < PO-PEG SPIONs < DSPE-PEG SPIONs. These differences vary in statistical significance, but this trend was evident across all samples.

Zeta potential (ζ) results also reflected interesting trends. Generally speaking, ζ decreased with increasing PEG chain length (for 4nm PO-PEG SPIONs, 4nm NH_2_-PEG SPIONs, and 10nm DSPE-PEG SPIONs). For 4nm DSPE-PEG SPIONs, ζ showed high error and no observable trend. For 10nm PO-PEG SPIONs, ζ showed low error, but no definitive trend. And for 10nm NH_2_-PEG SPIONs, ζ appeared to be bimodal, with 1k and 3.4k samples having more similar ζ values, which were different than the similar ζ values shared by 2k and 5k samples. Given the same PEG chain length and PEGylation method, ζ for 4nm samples were generally closer to neutral than ζ for the equivalent 10nm sample. A definitive trend in ζ as a function of PEGylation method was not observed.

TGA results generally indicated averages similar to the targeted surface density of 0.7 PEG chains/nm^2^. Importantly for comparison, there were no statistically significant differences (p > 0.05) in surface density between particles within the same PEGylation method and core size (e.g., all 10nm PO-PEG SPIONs) – with the exception of 4nm DSPE-PEG 5k samples, which exhibited a significantly higher surface density than either 1k or 2k DSPE-PEG samples of the same core size (p < 0.05). This suggests that we can attribute differences in MR relaxivities (described below) for particles with the same PEGylation method and core size to variations in PEG chain length, with the exception of results from the 4nm DSPE-PEG 5k samples. Similarly, there were no statistically significant differences in surface density between particles with the same PEG chain length and core size (e.g., all 4nm SPIONs with any 1k PEG). This suggests that we can attribute variations in MR relaxivities for particles with the same core size and PEG chain length to differences in PEGylation method and their resulting chemical differences.

**Figure 2.**
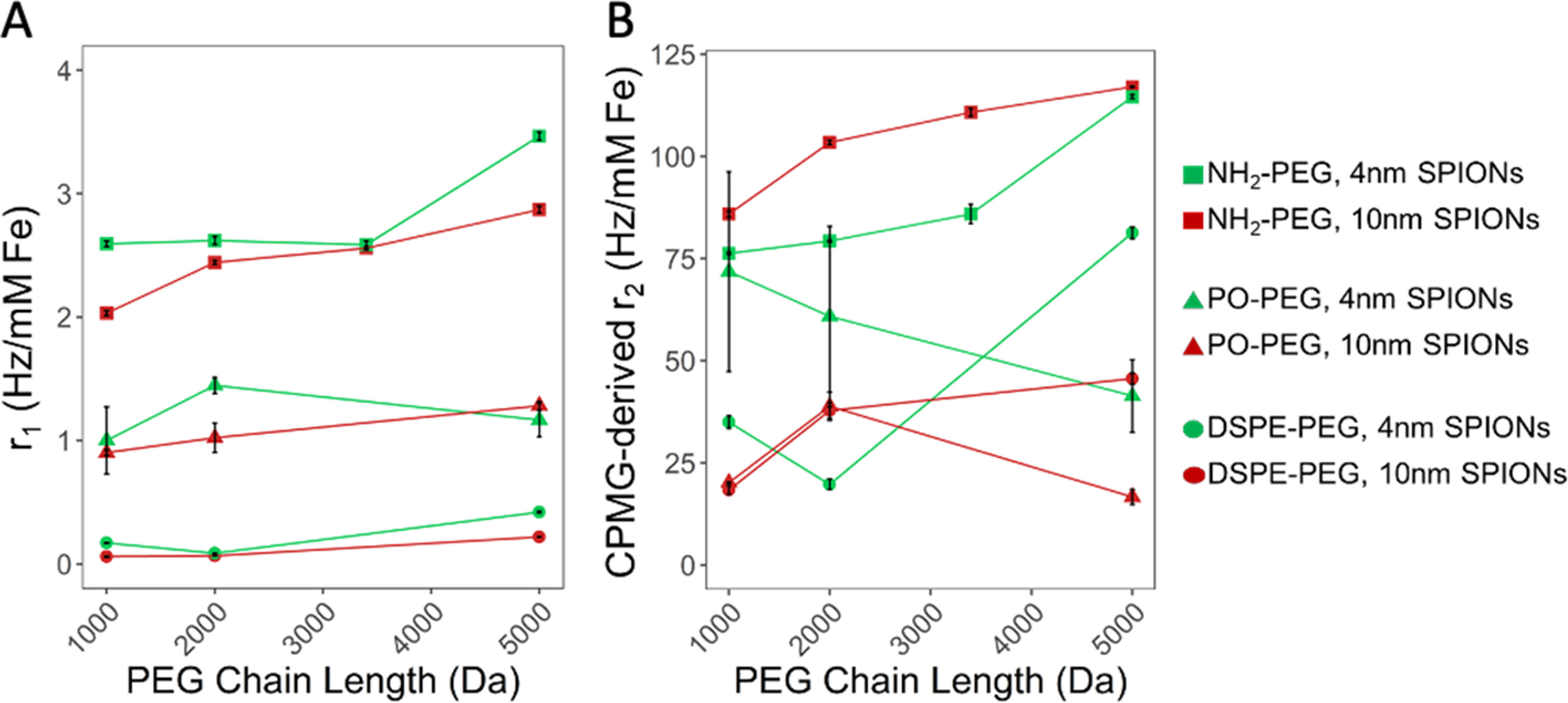
(A) Impact of SPION core size and PEG chain length on r_1_ for PO-PEG SPIONs, NH_2_-PEG SPIONs, and DSPE-PEG SPIONs. Error bars are standard error in the calculated slope value. (B) Impact of SPION core size and PEG chain length on r_2_ CPMG (see Supplemental Materials and Methods) for PO-PEG SPIONs, NH_2_-PEG SPIONs, and DSPE-PEG SPIONs. Error bars are standard error in the calculated slope value.

The impact of SPION core size, PEGylation method, and PEG chain length on r_1_ relaxivity was notable (Fig. 2A). DSPE-PEG SPIONS had markedly lower r_1_ relaxivity than either NH_2_- or PO-PEG SPIONs. For 4nm SPIONs with comparable PEG chain lengths, NH_2_-PEG SPIONs had r_1_ relaxivities at least 2.15x higher than PO-PEG SPIONs, and at least 8.23x higher than DSPE-PEG SPIONs for equivalent PEG chain lengths. For 10nm SPIONs, NH_2_-PEG SPIONs had r_1_ relaxivities of at least 2.09x higher than PO-PEG SPIONs, and at least 33.87x higher than DSPE-PEG SPIONs.

Interestingly, for NH_2_- and PO-PEG SPIONs, the maximum effect imparted on r_1_ relaxivity by changing PEG chain length was between 34-45% and was not markedly different for the two different core sizes. However, for DSPE-PEG SPIONs, r_1_ relaxivity differed by a factor of 360 (10 nm core) to 480% (4 nm core) by changing PEG chain length.

For 10nm SPIONs, increasing the PEG chain length generally resulted in an increase in r_1_. For 4nm SPIONs, a trend was not apparent. With the notable exception of the 5k PO-PEG SPIONs (for which the 10nm SPION had a higher r_1_ relaxivity), for all PEGylation methods and PEG chain lengths, 4nm SPIONs had higher r_1_ relaxivities than their 10nm counterparts. This is the opposite effect than expected (and previously reported) for r_2_ relaxivity. Differences in otherwise equivalent samples with different core sizes ranged from 0.91x (PO-PEG5k, where the r_1_ of 10nm samples was greater than that of 4nm samples) to 2.84x (DSPE-PEG1k).

The impact of SPION core size, PEGylation method, and PEG chain length on r_2_ (CPMG method) was significant (Fig. 2B), and only partly in line with what was observed with the r_1_ trends. The PEGylation method and resulting chemical composition of the layer closest to the SPION core had the most definitive impact on r_2_ relaxivities. In line with r_1_ results, across PEG chain lengths, NH_2_-PEG SPIONs had the highest r_2_ relaxivities; 1.41–4.67x higher than DSPE-PEG SPIONs and 1.06–7.02x higher than PO-PEG SPIONs.

For NH_2_-PEG SPIONs with both 4nm and 10nm cores, increasing PEG chain length led to an increase in r_2_ relaxivity. This was also the case only for DPSE-PEG SPIONs with 10 nm cores. For DSPE-PEG SPIONs with 4nm cores the largest PEG length also led to the largest r_2_, but there was a drop in r_2_ at the intermediate PEG length. For PO-PEG SPIONs with 10 nm cores, there was not a definitive trend. However, for PO-PEG SPIONs with 4nm cores, increasing the PEG chain length led to a consistent decrease in r_2_ relaxivity. Additionally, for NH_2_-PEG SPIONs, r_2_ relaxivities were higher for the 10nm core sizes at each PEG chain length, with differences in equivalent samples with different core sizes ranging from 1.02x (PEG5k) to 1.30x (PEG2k). However, the opposite was true for PO-PEG SPIONs, where differences in equivalent samples with different core sizes ranged from 1.57x (PEG2k) to 3.56x (PEG1k). Differences in equivalent samples with different core sizes for DSPE-PEG SPIONs did not show a consistent trend.

**Figure 3.**
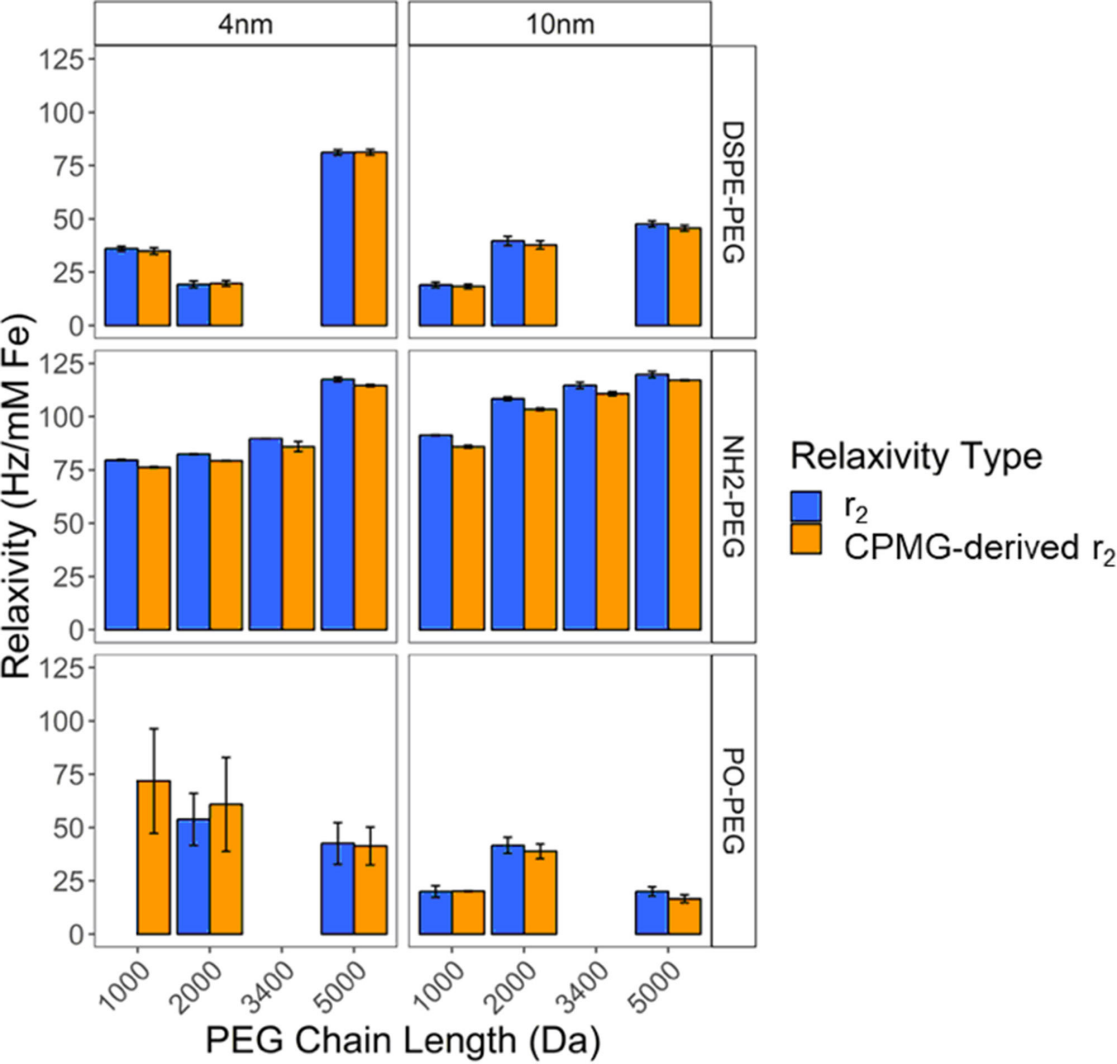
Comparison between relaxivities derived from spin echo (SE) measurements (blue) and CPMG measurements (orange). The general similarity of these results indicates that diffusion of particles in solution did not play a substantial role in altering measured relaxation rates. Error bars are standard error in the calculated slope value. For a particular data set (the 1k PO-PEG SPIONs with a 4nm core), r_2_ rates are not reported for the SE method, as that method is even less robust to the presence of inhomogeneities, referred to in the Supplementary Methods and Materials.

Spin echo-derived r_2_ relaxivities were higher than or comparable to (i.e., calculated values share an overlapping standard error range) CPMG relaxivities as expected (Fig. 3). Where they were greater, r_2_ SE-derived relaxivities were 1.023x to 1.20x the r_2_ CPMG-derived relaxivities, with the highest ratio observed for 5k PO-PEG, 10nm SPIONs.

**Figure 4.**
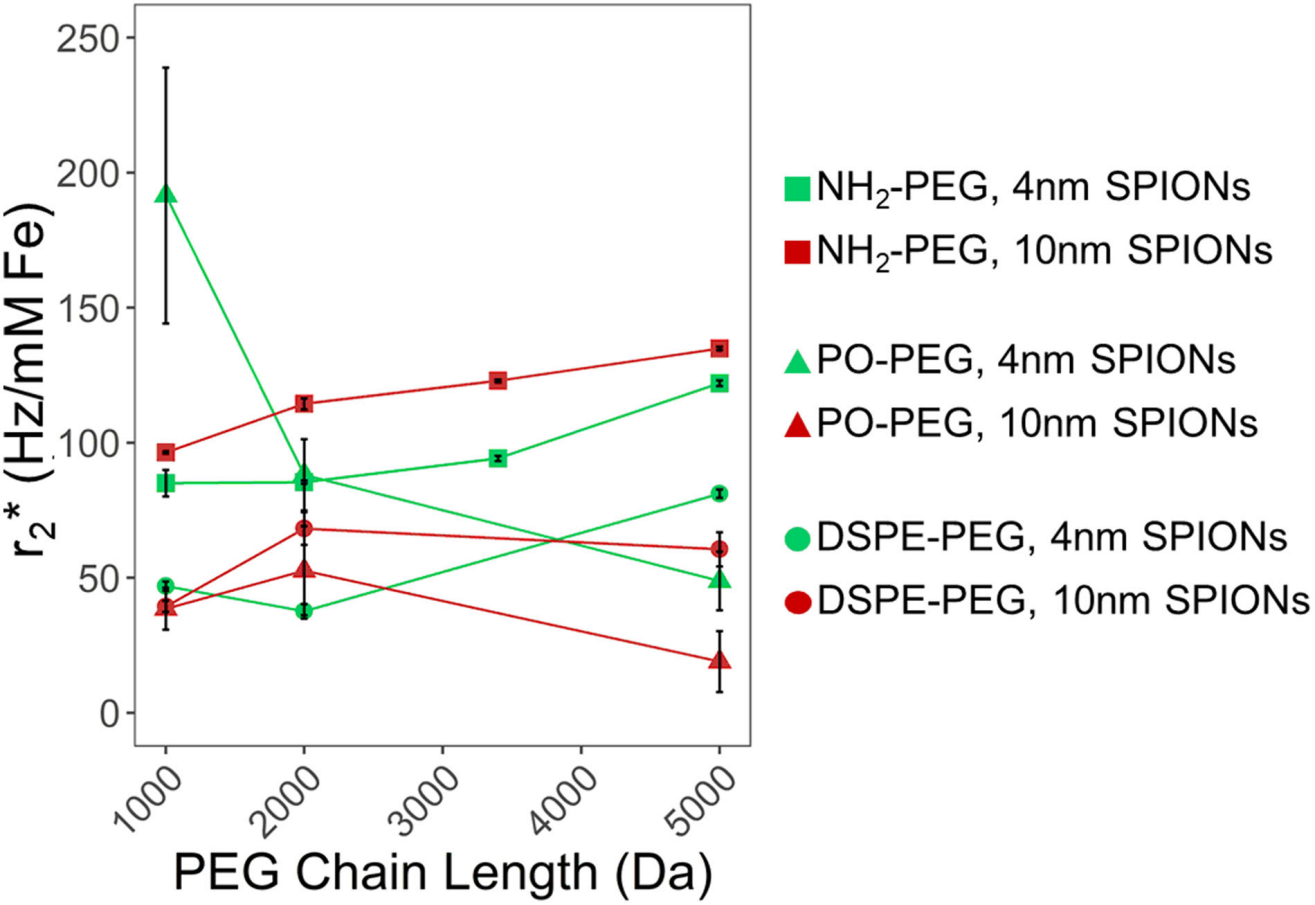
Impact of SPION core size and PEG chain length on r_2_* for PO-PEG SPIONs, NH_2_-PEG SPIONs, and DSPE-PEG SPIONs. Error bars are standard error in the calculated slope value.

With the exception of 4nm 1k PO-PEG SPIONs, NH_2_-PEG SPIONs had the highest r_2_* relaxivities across PEG chain lengths (Fig 4). For NH_2_-PEG SPIONs with both 4nm and 10nm cores, increasing PEG chain length led to an increase in r_2_* relaxivity. This was also the case only for DPSE-PEG SPIONs with 10nm cores. For DSPE-PEG SPIONs with 4nm cores, and for PO-PEG SPIONs with 10nm cores, there was not a definitive trend. However, for PO-PEG SPIONs with 4nm cores, increasing the PEG chain length led to a consistent decrease in r_2_* relaxivity. Additionally, for NH_2_-PEG SPIONs, r_2_* relaxivities were higher for 10nm core sizes at each PEG chain length, while the opposite was true for PO-PEG SPIONs. Differences in equivalent samples with different core sizes for DSPE-PEG SPIONs did not show a consistent trend.

**Figure 5.**
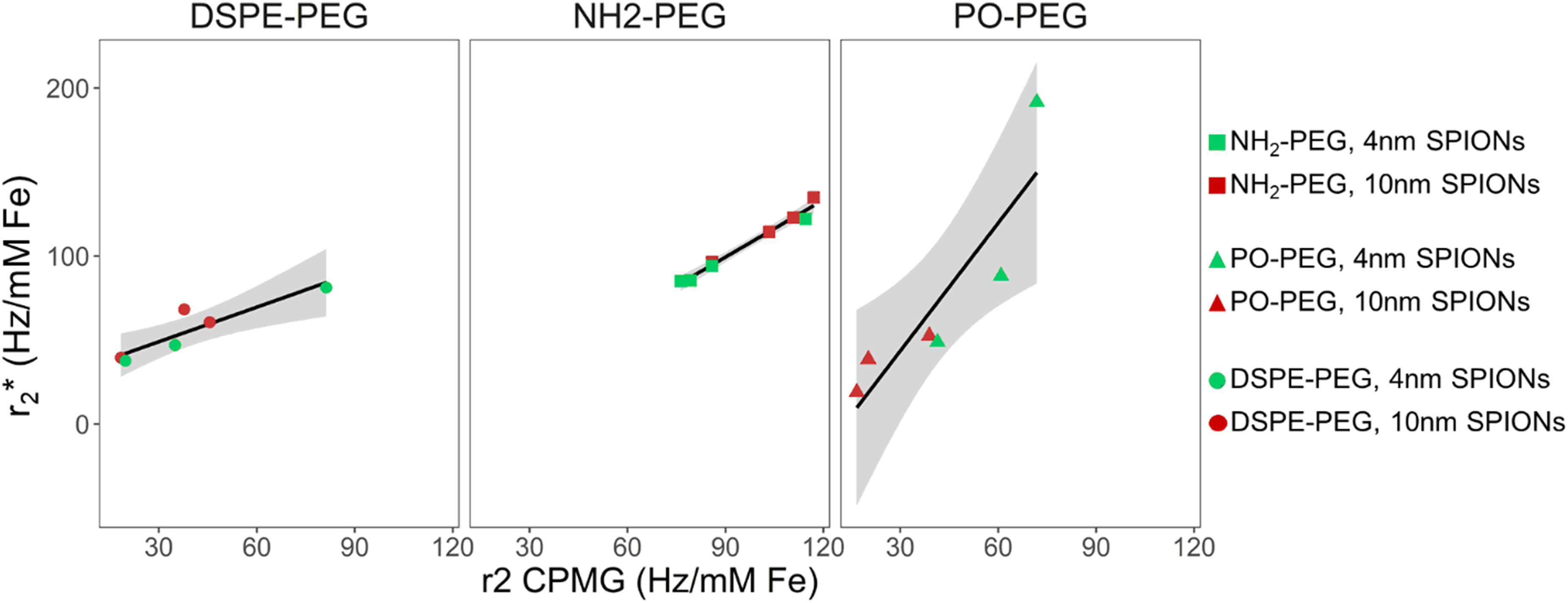
Relationship between r_2_* and CPMG-derived r_2_ values across all samples. Black lines indicate fit from linear regression; grey shading indicates 95% confidence interval. There is a strong positive correlation between r_2_* and r_2_ for each PEG attachment method (all R > 0.85). For NH_2_-PEG SPIONs: r_2_* vs r_2_ (R = 0.986). Slopes of linear regression: 0.69 (DSPE-PEG), 1.13 (NH_2_-PEG), and 2.54 (PO-PEG).

As expected, r_2_* vs. r_2_ relaxivities for each given sample were either comparable to (with inclusion of standard error) or significantly greater than CPMG relaxivities (Fig. 5). Where they were greater, r_2_* were 1.08x to 2.67x the r_2_ CPMG-derived relaxivities, with the highest ratio observed for 1k PO-PEG, 4nm SPIONs.

## III. Discussion

It would generally be expected that, with equivalent core sizes and PEGylation methods, longer PEG chain lengths would lead to larger effective diameters, as observed with PO-PEG SPIONs (Table 1). However, in cases where this was not observed, it is possible that aggregation effects were at play. This seems to be a likely explanation particularly for DSPE-PEG SPIONs, as the effective diameter of 5k DSPE-PEG SPIONs was generally substantially lower than 2k DSPE-PEG SPIONs of the same core size. The larger standard deviation observed in DSPE-PEG SPION effective diameter measures could also be reflective of aggregation. For NH_2_-PEG SPIONs, where the general size progression is 1k < 2k < 5k < 3.4k, the observed difference between 3.4k and 5k samples may be due to a difference in the preferred PEG chain conformation, or potentially to a small amount of aggregation occurring, as the standard deviations are not as high as those observed for DSPE-PEG SPIONs.

In comparing samples with similar PEGylation methods and PEG chain lengths, it would be expected that the effective diameter of 4nm samples would be smaller than 10nm samples. This was confirmed for both PO-PEG and NH_2_-PEG SPIONs. However, this was not true for DSPE-PEG SPIONS. It is likely that, again, aggregation plays a role in these results.

Zeta potential measurements reflect the potential at the slipping/shear plane of particles in solution [31]. While PEG itself does not carry a high surface charge, SPIONs alone in solution tend to carry a high negative charge (e.g., CA-SPIONs have a ζ ∼−35 mV). It is thus expected that coating with the more neutral PEG will raise the ζ of the particles closer to neutral (0mV) [31], [32]. This was observed in all cases.

The general increase towards neutral of ζ with increasing PEG chain length may be expected; it is possible that longer PEG chains help to better “mask” the highly negatively charged SPION core. The higher ζ in 4 nm samples as compared to 10 nm samples of the same PEGylation method and PEG chain length may be due to the same effect. Finally, it is intuitively understood that there was no observed trend in ζ as a function of PEGylation method, since solution-facing ends of each PEG chain were chemically identical regardless of PEGylation method.

The observed results from TGA tests indicated that the majority of samples have non-significant differences in terms of surface coverage (with the exception of 4nm DSPE-PEG 5k samples, which have a statistically significantly greater surface coverage). This indicates that, with the exception of 4nm DSPE-PEG 5k SPIONs, observed differences in MR relaxivities between samples will solely be due to differences in core size, PEG chain length, or PEGylation method. This is an important confirmation step that has been omitted in previous literature examining the impact of these parameters on SPION MR properties [15], [29]. We also note that all surface densities are well above the critical surface density required for overlapping chains [33].

Perhaps the most salient trend in MR measurements is that r_1_ and r_2_ relaxivities are greater in NH_2_-PEG SPIONs than either PO-PEG or DSPE-PEG SPIONs of the same core size and PEG chain length. It is hypothesized that this is due to the hydrophilic nature of the group closest to the core for these SPIONs (citric acid), in comparison to the less hydrophilic nature of the phosphine oxide and the hydrophobic nature of the DSPE chains. The more hydrophilic group near the core may allow water to move more easily closer to the core and spend more time in proximity to the magnetic field generated by the core. In comparing PO-PEG and DSPE-PEG SPIONs across particles with the same core size and PEG chain length, results appear to differ between r_1_ and r_2_ relaxivities. PO-PEG SPIONs show far higher r_1_ relaxivities across the board. However, the difference between r_2_ relaxivities for PO-PEG and DSPE-PEG SPIONs do not show a consistent trend.

For NH_2_-PEG and DSPE-PEG SPIONs, within a given SPION core, increasing PEG chain length from 1k to 5k generally led to an increase for r_1_ and CPMG-derived r_2_ relaxivities. This is consistent with results from Tong, et al. who considered PEG chain lengths over 1kDa [29]. This is likely because increased PEG chain length creates a larger corona, slowing movement of water molecules around the particle, thus increasing the amount of time water protons spend in proximity to the core. Interestingly, this is not true for PO-PEG SPIONs, which do not show a consistent trend with PEG chain length.

Results of this study do not necessarily corroborate some previous findings, [4], [27] i.e., SPIONs with larger core sizes yield higher r_2_ relaxivities in samples with equivalent PEG chain length and PEGylation method. Although this is true for NH_2_–PEG SPIONs, there is a less distinctive trend for both PO-PEG and DSPE-PEG SPIONs. The trend observed in 5kDa PO-PEG SPIONs could potentially be linked to the increased surface coverage of 4nm samples as measured by TGA; however, this is not the case in 1kDa samples. On the other hand, SPIONs with larger core sizes generally yielded lower (or comparable) r_1_ relaxivities in samples with equivalent PEG chain length and PEGylation method. This trend may be related to recent results demonstrating that SPIONs with sufficiently small core diameters can be used as T_1_ contrast agents [34], [35]. Additional work is needed to further elucidate the impact of SPION core size on MR relaxivities with different PEG attachment methods.

## IV. Conclusion

Our results suggest the chemical composition and, in particular, hydrophobicity/hydrophilicity of the group linking PEG chains to a SPION core may play a larger role in the resulting MR relaxivities than other variable properties such as SPION core size and PEG chain length. In addition, impact of SPION core size and PEG chain length on the resulting sample relaxivities is not a simple relationship, but appears to be conflated with the impact of PEGylation method and nature of the chemical group closer to the SPION core.

Such comprehensive and controlled studies of the impact of different fabrication parameters on MR relaxivities are necessary to provide a framework to optimize SPION MR properties and reveal the complexity of interactions involved in SPION functionalization. This work carries implied clinical significance in terms of the development of future MR contrast agents with properties that may be beneficial for the diagnosis and safety of patients.

## V. Materials and Methods

A number of SPIONs were synthesized to be physiochemically distinct in terms of their core size, PEG chain length, and the method of PEG attachment to the SPION core. SPION properties including size, zeta potential, and coating density of the attached PEG were evaluated. The MR relaxivities of these SPIONs were subsequently measured and compared.

## Supplementary Materials

For specific Materials and Methods information, please refer to the Supplementary Materials, which contain a thorough description of all methods and materials used for the experiments described in this manuscript.


