# Supplementary material for: Assessment of Superparamagnetic Iron Oxide Nanoparticle Poly(Ethylene Glycol) Coatings on Magnetic Resonance Relaxation for Early Disease Detection
Source: IEEE Open J Eng Med Biol. 2020 May 4;1:116–22. doi: 10.1109/OJEMB.2020.2989468 (PMC7720857; doi:10.1109/OJEMB.2020.2989468)
Supplement: Supplementary file 1 [file supp1-2989468.pdf]

Assessment of Superparamagnetic Iron Oxide Nanoparticle Poly(Ethylene Glycol) Coatings Magnetic Resonance Relaxation for Early Disease Detection

Cari L. Meisel, Polly Bainbridge, Robert V. Mulkern, Dimitrios Mitsouras, and Joyce Y. Wong

I. Materials and Methods

SPION core sizes of 10 and 4nm were chosen to reflect those sizes that are often developed for MR applications in the literature.[1]–[3] The three PEGylation methods selected represent a cross section of different PEGylation methods widely used in the literature. It was hypothesized that the hydrophilicity or hydrophobicity of the chemical group closest to the SPION core would have a large impact on the MR relaxivities, as this is largely dependent on the ability of water to move around and close to the SPION cores.

A number of SPIONs were synthesized to be physiochemically distinct in terms of their core size, PEG chain length, and the method of PEG attachment to the SPION core. SPION properties including size, zeta potential, and coating density of the attached PEG were evaluated. The MR relaxivities of these SPIONs were subsequently measured and compared.

Method 1 (PO-PEG) is a direct ligand exchange method, where the SPION cores are PEGylated in a one-step process that relies on direct interaction between a functional group on the PEG and the SPION core.[4] This particular method will result in SPIONs with a small, combination hydrophilic/hydrophobic group closest to the core; PO-PEG dissolves readily in a combination of ethanol and n-hexane. Method 2 (NH_2-PEG) is a two-step method, where a ligand exchange is performed to first replace the original (often hydrophobic) coating of the SPIONs, followed by PEGylation via attachment to the new coating material.[5], [6] This particular method will result in SPIONs with a small, strongly hydrophilic group closest to the core. Method 3 (DSPE-PEG) is a one-step method that relies upon somewhat weaker hydrophobic interactions or associations to link the PEG to the SPION core.[7] This particular method will result in SPIONs with a bulky, very hydrophobic group closest to the core.

A. Materials

Iron tri(acetylacetonate) (2 mmol), 1,2-tetradecanediol (10 mmol), oleic acid (6 mmol), oleylamine (6 mmol), benzyl ether, citric acid, diethyl ether, 2-methoxyethylamine, N-(3-Dimethylaminopropyl)-N′-ethylcarbodiimide hydrochloride (EDC), N-Hydroxysuccinimide (NHS), 1,2-dichlorobenzene, and N,N’-dimethylformamide were all purchased from Sigma-Aldrich (St. Louis, Missouri). NH_2-PEG and 1,2-Distearoyl-sn-glycero-3-phosphoethanolamine (DSPE) – PEG reagents were obtained from Nanocs (Boston, Massachusetts); phosphine oxide (PO)-PEG reagents were obtained from JenKem Technology (Plano, Texas). Slide-A-Lyzer dialysis cassettes were acquired from Thermo Scientific (Rockford, Illinois). Syringe tip filters were obtained from Fisher Scientific (Waltham, Massachusetts). Cryogenic vials for sample measurements were obtained from Corning (Corning, New York).

B. Iron Oxide Particle Synthesis

Oleic acid-coated iron oxide nanoparticles were synthesized via thermal decomposition as previously described.[5], [6] Briefly, the following reagents were mixed and stirred magnetically under nitrogen flow: 2mmol iron(III) tri(acetylacetonate), 10mmol 1,2-tetradecanediol, 6mmol oleic acid, 6mmol oleylamine, and benzyl ether. The resulting nanoparticles were precipitated in excess ethanol and pulled down using a magnet. The precipitated particles were then washed with ethanol, dissolved in hexane, and centrifuged to remove aggregates. The SPIONs, which at this point are oleic acid-coated (abbreviated OA-SPIONs henceforth), were finally dried in a vacuum oven.

C. PEGylation

PEGylation of OA-SPIONs was accomplished via one of three protocols similar to the majority of those that have been previously used to functionalize SPIONs.

1) PO-PEGylation:[4] OA-SPIONs were dissolved in 1mL of n-heptane or 1mL of 1,2 dichlorobenzene while PO-PEG (molecular weight 1, 2, or 5 kDa) was dissolved in 1mL of ethanol. Sufficient PEG was used to target a coating density

of 0.70nm^{-2}. The mixture was heated (70˚C) and left stirring for at least 5 hours. PO-PEG coated nanoparticles (PO-PEG-SPIONs) were precipitated in n-hexane and collected on a magnet. They were then re-dispersed in ethanol before dispersing in deionized (DI) water and remaining ethanol was allowed to evaporate. Excess PEG was removed by dialysis for at least 48 hours against DI water.

2) NH_2-PEGylation:[5], [6] A ligand exchange was performed to replace the oleic acid with citric acid. Oleic acid-coated nanoparticles and citric acid were dissolved in a 1:1 mixture of dichlorobenzene and N,N-dimethylformamide. The mixture was heated to 100˚C and left stirring for 24 hours. Citric acid coated nanoparticles (CA-SPIONs) were precipitated in diethyl ether and washed with acetone and diethyl ether before drying in a vacuum oven.

For PEGylation, CA-SPIONs were dissolved in pH 9 sodium phosphate buffer and filtered using syringe PTFE filters (0.2 µm). NH_2-PEG (molecular weight 1, 2, or 5 kDa) was added to the CA-SPIONs in quantities sufficient to target a coating density of 0.70nm^{-2}. 1-Ethyl-3-(3-dimethylaminopropyl)carbodiimide (EDC) and N-hydroxysuccinimide (NHS) were added in molar excess to the solution, which was allowed to mix overnight at room temperature. Excess PEG was removed by dialysis for at least 48 hours against DI water.

3) DSPE-PEGylation:[7] OA-SPIONs were dissolved in chloroform. A corresponding polymer solution was created through the dissolution of DSPE-PEG (molecular weight 1, 2, or 5 kDa) in a 1:1 mixture of chloroform and acetonitrile in quantities sufficient to target a coating density of 0.70nm^{-2}. The two solutions were mixed for 15 minutes while 10mL of water and two washed marbles were warmed to 70°C. A rotary evaporator was used to remove the chloroform and acetonitrile, creating a thin film of particles and PEG on a roundbottom flask. The water and marbles were then added to the flask and swirled vigorously for 15 minutes until the film dissolved. Once mixed, the solution was filtered through a 0.2µm syringe filter. DSPE-PEGylated nanoparticles were separated from DSPE-PEG micelles or liposomes by pulling down onto a magnet. The isolated DSPE-SPIONs were re-dispersed in water.

D. DLS and Zeta Potential Measurements

DLS measurements were chosen to observe effective rather than absolute diameters as the behavior of particles in solution was of particular interest for this study. Differences in the amount of aggregation or the spread of the PEG layer should be reflected in differences in the measured effective diameter. Because both aggregation as well as PEG chain conformation may impact measured MR relaxivities, effective diameter measurements were of more use than absolute diameter as measured by methods such as, e.g., transmission electron microscopy (TEM).

The effective diameter of all SPIONs produced was measured via dynamic light scattering (DLS) in water. Samples were diluted 10x in water and measured on a 90Plus DLS Analyzer (Brookhaven Instruments) at a 90˚ detection angle. Dilution was performed because previous studies have indicated that high concentrations of nanoparticles may disrupt DLS readings.[8] Zeta potential measurements were performed on the same samples using the same apparatus.

E. Thermogravimetric Analysis

The density of polymer coating was measured by thermogravimetric analysis (TGA) using a Discovery TGA system (TA Instruments). Samples were lyophilized to remove all water, and at least 1mg of each sample for each run was loaded onto a TGA pan. All TGA analysis was performed under nitrogen flow. Samples were ramped to 100°C and allowed to equilibrate to remove any additional water that may have been absorbed by samples during storage. Then, samples were ramped to 650°C and allowed to equilibrate. The mass remaining at the end of this ramping step was considered to be solely comprised of the iron oxide core of the particles; these values were used to calculate the amount of PEG that was attached to the particles prior to TGA analysis, and the corresponding surface density of PEG on each sample.

F. Preparation of Samples for MR Measurements

The iron concentration in each resulting solution containing PEGylated particles was determined using a simple absorbance assay at 510nm, based on solutions containing known quantities of iron. Solutions at different concentrations (0, 5, 25, 50, 75, and 100 ppm) were prepared by dilution in water for MR. The solutions were stored in plastic cryogenic vials, and the amount of air in each vial was minimized to eliminate signal interference from the air-water interface. All vials were embedded into Styrofoam for MR measurements.

G. MR Equipment and Experiments

Four MR experiments were performed for each set of samples. Two experiments were designed to measure R_2, one was designed to measure R_2^*, and one was designed to measure R_1. All experiments were performed on a 3 Tesla GE HDx clinical MR imager (GE, Milwaukee, WI) equipped with 40 mT/m gradients capable of 150 mT/m/sec slew rate and an 8 channel brain phased array receiver coil for signal reception.

The first experiment was designed to measure R_2 using a two-dimensional “Hahn” single echo spin echo sequence with a repetition time (TR) of 2 sec and repeated independently for 11 echo times (TE) ranging from 9 ms to 125 ms (TE_i =9, 14, 20, 26, 34, 42, 50, 65, 80, 102.5 and 125 ms). Other sequence parameters were 2 mm slice thickness, 13-15 cm field-of-view (FOV), 256×256 matrix, 2 signal averages and ±19 kHz bandwidth. With these parameters, a typical signal-to-noise ratio (SNR) was 40 at the shortest TE,

reducing to 10 no earlier than the fourth echo for the highest agent concentrations.

Because T2 measurements using this Hahn single echo spin echo sequence are sensitive to molecular diffusion, a second experiment was performed to measure R_2 using a three-dimensional multi-echo spin echo Carr-Purcell-Meiboom-Gill (CPMG) sequence.[9]–[13] This sequence utilized non-selective radiofrequency pulses with composite refocusing (90_x-180_y-90_x) as well as gradient crushers that alternated in polarity at every echo and that diminished in amplitude at every pair of echoes.[14] Sequence parameters were 1.5 sec TR, 16 echoes evenly spaced at 7.4 ms (first echo at 7.4 ms, last echo at 118.4 ms), 2 mm slice thickness, 13-15 cm FOV, 96 (readout) × 72 (phase) × 38 (slice) matrix, 1 signal average, and ±32.8 kHz bandwidth. SNR was typically >100 at the first echo, reducing to no less than 30 at the 4th echo for the highest agent concentration.

R_1 was measured using a two-dimensional single echo spin echo sequence with a fixed echo time of 9 ms and independently repeated for 12 repetition times ranging from 100 ms to 9 sec (TR_i=100, 250, 500, 750, 1000, 1366, 1750, 2500, 4000, 5500, 7000, and 9000 ms). Other parameters were identical to the single echo spin echo R_2 experiment except that 4 signal averages were used for the shorter TRs in order to increase SNR. For acquisitions with TR >2.5 sec, only 1 signal average was used to maintain a reasonable scan time. With these parameters, SNR was between 10-30 for the three shortest TRs and >30 for the remaining TRs.

Finally R_2^* was measured using a two-dimensional multi echo gradient recalled echo sequence with a 1.5 sec TR and 8 echoes collected between 3.6 and 40 ms with a spacing of 5.3 ms. Other sequence parameters were 2 mm slice thickness, 13-15 cm FOV, 256 × 256 matrix, 2 signal averages and ±32.8 kHz bandwidth. SNR was 12 at the first echo and dropped to approximately 3 by the 4th echo at the highest agent concentration.

H. MR Analysis

Analysis of MR signal intensities to determine sample relaxation rates was performed using custom software developed in MATLAB (version R2013b, Mathworks Inc, Waltham MA). The software performed non-linear non-negative least squares fitting of signal intensity measurements across either the echo (for T2, $T2^*$) or the repetition times (for T1), using idealized mono-exponential signal models. For T2 and $T2^*$ measurements, the signal model was $s(TE_i) = s_{TE=0}e^{-TE_iR}$, where R the corresponding R_2 or R_2^* relaxation rate. For T1 measurements, the signal model was $s(TR_i) = s_{TR=\infty}(1 - e^{-TR_iR_1})$. In both models, the non-linear optimization routine solved for the spin density $s_{t=\{0,\infty\}}$ and the corresponding relaxation rate.

Signal intensities in each sample were measured using a custom software (also developed in MATLAB). The software allowed all DICOM images corresponding to a single experiment (e.g., all spin echo acquisitions with a single TR and varying TE for a T2 experiment) to be loaded, and regions-of-interest (ROIs) to be placed in any source axial slice in the image volume of one acquisition. The software then propagated the ROI to the corresponding slice and location in every acquisition (e.g., the multiple TEs or multiple TRs) in the experiment, thereby producing the average signal measurement for that ROI across the corresponding independent variable. ROIs were placed at the center of each vial for each solution concentration in at least 4 different axial slices covering the vial. The fitted relaxation times were recorded into Excel and averaged over the multitude of ROIs collected for each vial. A least squares linear fit was finally used to calculate the agent relaxivity (r_1, r_2, r_2^*) as the slope of a straight line corresponding to the measured relaxation rates (R_1, R_2, R_2^*) vs. known iron concentration values of each vial. Standard error in the slope was also estimated from this output. For a small number of data sets that had increased artifact due to significant B_0 and/or B_1 inhomogeneity, i.e., where incomplete refocusing could not be ruled out even with the use of composite radiofrequency refocusing pulses in the CPMG sequence, ROIs were more carefully selected for analysis based on agreement of the fitted R_2 values from CPMG signal decays with an additional acquisition that concurrently characterizes both the reversible and irreversible transverse relaxation rates in the presence of different intravoxel resonance distributions.[15]

Plotting of all the results was done in R (version 3.5.1) using ggplot2.

REFERENCES

[1] R. Jin, B. Lin, D. Li, and H. Ai, “Superparamagnetic iron oxide nanoparticles for MR imaging and therapy: design considerations and clinical applications,” *Curr. Opin. Pharmacol.*, vol. 18, pp. 18–27, Oct. 2014.

[2] Wahajuddin and S. Arora, “Superparamagnetic iron oxide nanoparticles: magnetic nanoplatforms as drug carriers,” *Int. J. Nanomedicine*, vol. 7, pp. 3445–3471, 2012.

[3] M. Di Marco, C. Sadun, M. Port, I. Guilbert, P. Couvreur, and C. Dubernet, “Physicochemical characterization of ultrasmall superparamagnetic iron oxide particles (USPIO) for biomedical application as MRI contrast agents,” *Int. J. Nanomedicine*, vol. 2, no. 4, pp. 609–622, Dec. 2007.

[4] B. H. Kim *et al.*, “Large-Scale Synthesis of Uniform and Extremely Small-Sized Iron Oxide Nanoparticles for High-Resolution T1 Magnetic Resonance Imaging Contrast Agents,” *J. Am. Chem. Soc.*, vol. 133, no. 32, pp. 12624–12631, Aug. 2011.

[5] Y. C. Park *et al.*, “Effect of PEG Molecular Weight on Stability, T2 contrast, Cytotoxicity, and Cellular Uptake of Superparamagnetic Iron Oxide Nanoparticles (SPIONs),” *Colloids Surf. B Biointerfaces*, vol. 119, pp. 106–114, Jul. 2014.

[6] Y. Park *et al.*, “Stability of Superparamagnetic Iron Oxide Nanoparticles at Different pH Values: Experimental and Theoretical Analysis,” *Langmuir*, vol. 28, no. 15, pp. 6246–6255, Apr. 2012.

[7] S. Tong, S. Hou, Z. Zheng, J. Zhou, and G. Bao, “Coating Optimization of Superparamagnetic Iron Oxide Nanoparticles for High T2 Relaxivity,” *Nano Lett*, vol. 10, no. 11, pp. 4607–4613, 2010.

[8] J. Szydlowski and W. A. Van Hook, “Concentration and Temperature Dependence of Dynamic Light Scattering for Some

Polystyrene Solutions in Toluene, Cyclohexane, Methylcyclohexane and Deuteromethylcyclohexane," *Macromolecules*, vol. 31, no. 10, pp. 3266–3274, May 1998.

[9] D. Mitsouras, R. V. Mulkern, and S. E. Maier, "Multi-Component T2 Relaxation Studies of the Avian Egg," *Magn. Reson. Med.*, vol. 75, no. 5, pp. 2156–2164, May 2016.

[10] D. Mitsouras *et al.*, "Immobilized Contrast Enhanced (ICE) MRI: Gadolinium-based long-term MR Contrast Enhancement of the Vein Graft Vessel Wall," *Magn. Reson. Med. Off. J. Soc. Magn. Reson. Med. Soc. Magn. Reson. Med.*, vol. 65, no. 1, pp. 176–183, Jan. 2011.

[11] D. Mitsouras, R. V. Mulkern, and F. J. Rybicki, "Strategies for inner volume 3D fast spin echo magnetic resonance imaging using nonselective refocusing radio frequency pulsesa)," *Med. Phys.*, vol. 33, no. 1, pp. 173–186, Jan. 2006.

[12] D. Mitsouras *et al.*, "Three-dimensional printing of MRI-visible phantoms and MR image-guided therapy simulation., 3D Printing of MRI-Visible Phantoms and MR Image-Guided Therapy Simulation," *Magn. Reson. Med. Magn. Reson. Med.*, vol. 77, 77, no. 2, 2, pp. 613, 613–622, Feb. 2017.

[13] R. Mulkern *et al.*, "Lung Parenchymal Signal Intensity in MRI: A Technical Review with Educational Aspirations Regarding Reversible Versus Irreversible Transverse Relaxation Effects in Common Pulse Sequences," *Concepts Magn. Reson. Part Bridg. Educ. Res.*, vol. 43A, no. 2, pp. 29–53, Mar. 2014.

[14] C. S. Poon and R. M. Henkelman, "Practical T2 quantitation for clinical applications," *J. Magn. Reson. Imaging*, vol. 2, no. 5, pp. 541–553, Sep. 1992.

[15] R. V. Mulkern, M. Balasubramanian, and D. Mitsouras, "On the lorentzian versus Gaussian character of time-domain spin-echo signals from the brain as sampled by means of gradient-echoes: Implications for quantitative transverse relaxation studies," *Magn. Reson. Med.*, vol. 74, no. 1, pp. 51–62, Jul. 2015.
